# Team cognition in healthcare simulation: a framework for deliberate measurement

**DOI:** 10.1186/s41077-025-00333-7

**Published:** 2025-03-24

**Authors:** J. Colin Evans, M. Blair Evans, Lorelei Lingard

**Affiliations:** 1 Division of Emergency Medicine, Schulich School of Medicine and Dentistry, Western University, London, ON Canada; 2 Department of Psychology, Western University, London, ON Canada; 3 Department of Medicine, Centre for Education Research and Innovation, Schulich School of Medicine and Dentistry, Western University, London, ON Canada

**Keywords:** Healthcare simulation, Healthcare teams, Team training, Team cognition, Mental model, Situational awareness, Observation, Nontechnical skills, Action teams, Crisis resource management

## Abstract

**Introduction:**

Team mental models and team situational awareness are key components of healthcare team simulation. Human factors and organizational psychology researchers have developed clear definitions and theories about these concepts that are at times ‘lost in translation’ within the prevailing forms of measurement and training utilized in healthcare. Simulation research to date has often relied upon indirect and imprecise measures and a conceptualization of team cognition that ill equips simulation educators as they endeavour to optimize healthcare team performance.

**Methods:**

We present a narrative review that examines how team cognition is assessed in healthcare team simulation, critically consider assessment strategies described in key studies, and contrast them to advances in human factors and organizational psychology.

**Results:**

This study presents a framework that reconceptualizes how we measure team cognition in healthcare simulation along the matrices of directness and timing of evaluation. We pair this framework with a table that exemplifies extant measurement techniques and highlight how simulation educators may decide between different ‘types’ of assessment based upon their needs.

**Discussion:**

We offer recommendations for educators to consider capturing team cognition before, during, and after simulation. We also offer recommendations for researchers to develop tools that may be more readily applied across key settings.

**Conclusion:**

Here, we present a framework of team cognition for healthcare action teams that advances healthcare simulation to better align with human factors and organizational psychology literature. This work will guide healthcare simulation educators and researchers on their quest to optimize team performance through improved team cognition.

**Trial registration:**

None.

**Supplementary Information:**

The online version contains supplementary material available at 10.1186/s41077-025-00333-7.

## Introduction

Team cognition refers team members’ collective understanding of their team structure, team member roles, and their team’s shared objective. The term team cognition encompasses numerous states, called team representations, which include concepts like team situational awareness and team mental models. Whereas representations are the way we *think* about our team and our task, team cognition also includes processes that are the things we *do *to develop and maintain our team’s shared understanding. Given its role in coordinating nearly all team processes, many training programmes have eagerly integrated elements of team cognition into their curriculum. But despite many advances in simulation-based training for healthcare team performance, the medical simulation and education literature has yet to adopt an effective measurement framework for evaluating team cognition [1–3]. The primary challenge of team cognition measurement centres on how we gather information: Currently the dominant measures of team cognition in healthcare simulation occur post-task and feature a broad assessment of coordinating behaviours, rather than specific facets of team cognition.

Rosenman et al. [4] identified the current challenges with team cognition measurement by summarizing how measures capture behaviours that relate to, but provide an indirect vantage toward, team cognition:

Measures are often indirect, using subjective self-report questionnaires or behavioral assessments that measure the actions supporting the development of situational awareness. While such measures are useful, they do not capture the cognitive processes underlying clinical decision making [4] pp.197

These tools are described as indirect, or subjective, approaches because they are more likely to reflect an observer’s interpretation of antecedents or outcomes of team cognition, but not the actual content or sharedness of team members’ thinking. Such tools are often nested within broad non-technical skills assessments that result in assumptions about cognitive representations. Such an approach can miss the mark by focusing on performance instead of process and lacks the specificity required to adequately understand team cognition [1]. This approach to team cognition has resulted in interventions and investigations that fail to measure, fail to report measurement outcomes, or measure with inadequate or inappropriately applied tools [5, 6]. There are signs of progress, as researchers have identified gaps in the healthcare literature and the need for more effective measures [1–6]. However, no articles or reviews to date have classified the large volume of existing measures through a critical lens.

In this *methodological intersections* article, we present a narrative review examining the state of team cognition measurement in healthcare teams and introduce definitions for team cognition, team mental models, and team situational awareness that align with definitions held in organizational psychology and human factors. We then present a novel framework built around two dimensions: directness and timing. These two dimensions combine to distinguish six ‘types’ of team cognition measures that play a unique role in how we understand and evaluate healthcare team cognition. By building upon findings from our previous scoping review [6] and recent systematic reviews [5, 7, 8], we classify various measurement approaches in healthcare teams simulation. In our discussion, we explore established practices in human factors in organizational psychology as we critically reflect on the complexity associated with measuring team cognition in healthcare team simulation. Through our framework, we aim to assist researchers and simulation educators in gaining awareness of what their tools are *actually* capturing and how they may better use and develop measurement tools to support team training.

### Defining teams and team cognition

Team cognition is a foundational element of team member interdependence. Yet, effective team cognition is both particularly important and particularly challenging to develop in teams that form in ad hoc, time-limited scenarios with variable composition and few fixed members. Referred to as action teams [9–12] or variable role, variable personnel teams [13], these teams are defined by their multi-professional (e.g. physician, nurse, social worker) and often multidisciplinary (e.g. emergency medicine, surgery, anaesthesia) membership and by their limited duration performance. These action teams are often found in acute care settings, and their performance is contingent upon members’ capacity to enter the situation with a shared understanding and to manage and update their understanding in a dynamic environment. Members of such teams often have varying familiarity with each other and must communicate across boundaries based on status, education, or role. The challenges facing acute care action teams are also what makes training these teams so ideally suited to simulation.

Team cognition is ‘the knowledge-building processes and/or emergent mental representations characterizing the degree of convergence of team-related knowledge content and structure’ [14] pp.443. This definition of team cognition by Mohammed, et al. [14] represents an umbrella construct consisting of ten constituent processes and representations from a variety of different literatures. While Mohammed argues for a maturing of team cognition through consolidation of these literatures, thus far such a consolidation has not yet taken place. In healthcare teams research the elements of team cognition that are most examined in the literature are information sharing (process), and team situational awareness and team mental models (representations).

According to Mohammed a *process* such as information sharing may be observed in an action team’s effective use of situation reports, the resulting enhancement of team situational awareness is the team’s collective mental *representation* that results from that process. Processes are often easier to observe directly because they are something that members ‘do’. The representations are, meanwhile, held within the minds of individuals and are challenging to measure accurately.

The unifying theme spanning these processes and representations is the degree of sharedness and accuracy of understanding across team members. Optimal healthcare team cognition is reflected in situations where teammates are on the same page and collectively possess an ‘accurate’ understanding of the clinical situation (e.g. what has happened to a patient so far?), their team (e.g. what are the responsibilities for each member?), and the task (e.g. what are the priorities?). Within the body of healthcare teams research, the primary focus is on two representations that form the overarching basis upon which healthcare team cognition is measured and evaluated: team situational awareness and team mental models.

Team situational awareness has its origins in human factors research and reflects the most widespread feature of team cognition as studied in simulation literature. This construct involves a team’s shared understanding and interpretation of ongoing events including *perception* of elements within a dynamic environment or system, *comprehension* of the meaning associated with these observations, and *projection *of these findings to support anticipation and response to future events [15]. Team mental models, meanwhile, are a component of team cognition native to organizational psychology research that focuses on ‘knowledge structures held by members of a team that enable them to form accurate explanations and expectations for the task, and, in turn, to coordinate their actions and adapt their behaviours to demands of the task and other team members’ [16] pp.228. Reflecting on how mental models and situational awareness relate to action teams entering a clinical task, individual mental models for each member are informed by training and past experiences. Once an action team forms for a clinical task, however, *team* mental models ‘emerge’ and the degree to which they are shared informs team members’ capacity to project the needs of the team and to contribute meaningfully to the development and maintenance of team situational awareness.

The complexity and time-sensitive nature of these teamwork dynamics mean that measurement within simulation can be challenging. For instance, one critique of healthcare team cognition research is that it has traditionally relied upon an approach that measures situational awareness indirectly and as a component of a broader assessment of nontechnical skills [1, 4]. Authors often name an item or scale as representative of team cognition, but it is conflated with other constructs (e.g. situational awareness and coping with stress given combined score [17]) or it captures signals that team members are seeking situational awareness (e.g. leaders making requests for status updates [18]). In the following sections, we present a novel framework to characterize healthcare team cognition measures followed by examples where various measures have been leveraged in healthcare teams literature. We then highlight the diversity in measures, common challenges, and key innovations in this setting.

## Methods

**Fig. 1:**
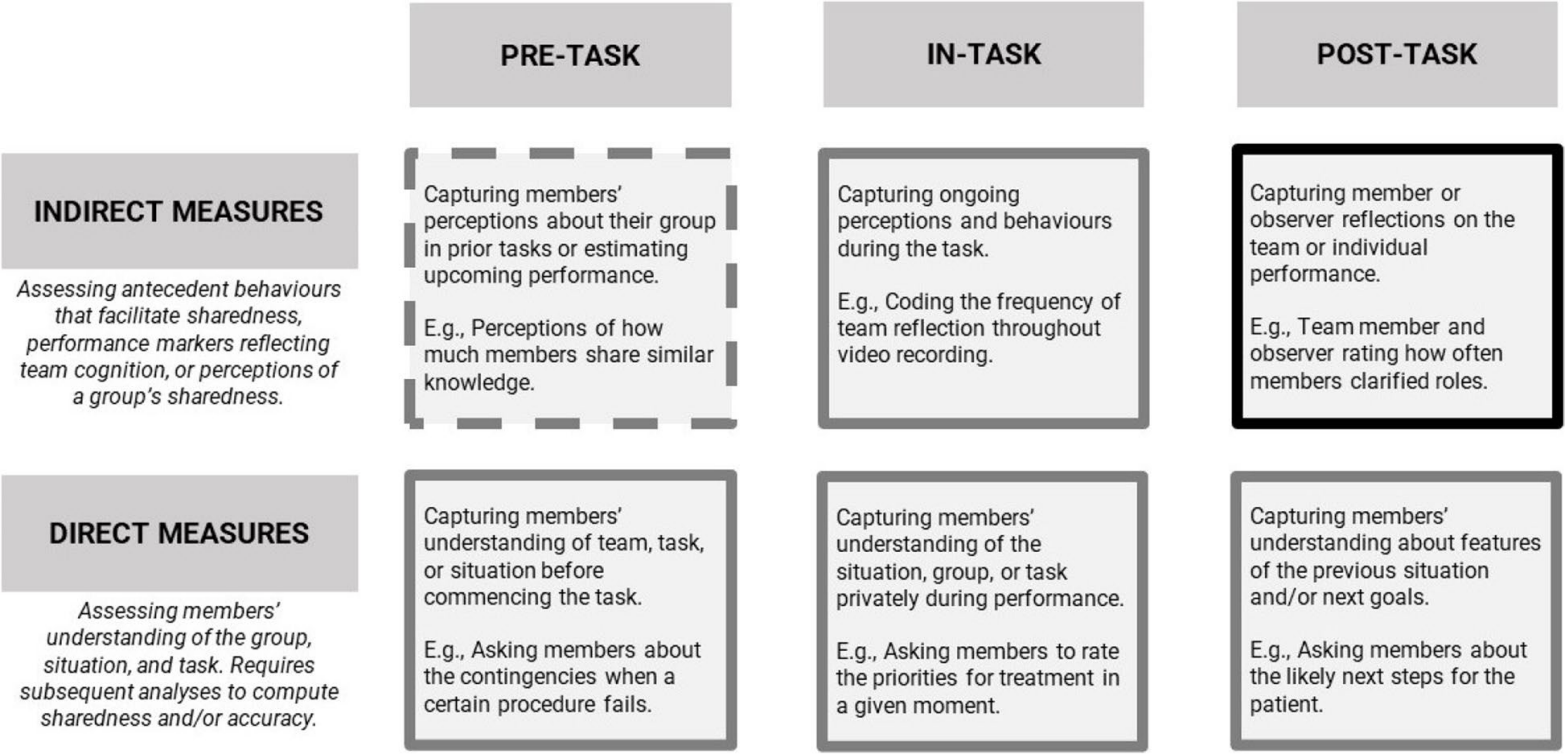
A framework for team cognition measure types. Six categories of team cognition measures as defined by timing and directness of measures. Indirect, post-task measures are highlighted with a darkened box because this is the measurement type dominated by the prevailing team cognition measure evidenced by tools such as NO-TECHS [18] and ANTS [25]. Remaining measure types are all reflected in the healthcare literature, with the exception of pre-task indirect measures (i.e. marked in a checkered box); despite being a plausible measure type, this was not identified in our review of cognition measures

We acknowledge that narrative reviews need not follow systematic review methodology (e.g. systematic search query, structured coding approach, risk-of-bias assessment) because they are intended to be configurative reflections of the literature by content experts. Indeed, our review did not include key markers of review methods. However, we structure our manuscript to include a methods and results section to define the scope of the literature incorporated as well as the process through which we created our framework for team cognition measurement. In our approach, we relied upon reviews in the organizational psychology and human factors literature [14, 19–24] to develop a comprehensive understanding of elements of team cognition measurement. Informed by these reviews, we generated a framework (Fig. 1) upon the axes of directness and timing of assessment tools. We then used this framework to identify examples of measures that have been operationalized in healthcare teams simulation. These examples were drawn first from a critical examination of recent systematic reviews of healthcare non-technical skills assessment [5, 7, 8] followed by a hand search of the literature to identify tools which fulfill categories of the framework that are less frequently examined. Through this method we generated a table of measures for team mental models and team situational awareness. For categories in which there were numerous exemplars we selected seminal tools upon which numerous subsequent iterations have been based. For categories in which options were more limited, we selected examples that most effectively present the method of tool deployment.

## Results

The novel framework presented in Fig. 1 highlights two prevailing dimensions across which team cognition measures fall: *how* team cognition is assessed (i.e. direct vs. indirect) and *when *it is assessed (i.e. pre-task, in-task, post-task). Within the healthcare literature, the prevailing form of measurement for team cognition falls into the indirect, post-task category of this framework. Studies presented in the human factors and organizational psychology literature highlight how observers often complete this type of measurement based more so on whether teams performed well rather than whether teams actually shared understanding [22]. Organizational psychology literature offers alternative types of evaluation that provide a more accurate understanding of how team cognition constructs are represented in the minds of team members and analysis techniques that can assess the sharedness of these constructs amongst the members of the team [14]. By collecting more direct data that captures the evolution of team cognition throughout their performance, we can generate more accurate feedback regarding the processes that support team cognition development [19].

Table 1 leverages our framework’s six categories of measures and then (a) provides brief descriptions of example measures (or families of measures) that are situated within that category and (b) highlights our observations about the source of data, operationalization, and specificity indirect and direct measures. Here, we highlight the diversity in measures based on what the ‘source’ of data is for a given measure (i.e. who provided raw data), the level and output of analyses (i.e. team or individual level construct computed), and the extent to which the measure was adapted for a context (e.g. specific medical specialty). It should be noted that these latter columns represent significant overlap between tools, and thus, operationalization and specificity are more overarching observations about the type of tools being used, and not one individual method.

**Table 1:** Characterizing direct and indirect team cognition measures [4, 18, 25–37]

**Tool type**		**Example measures**	**Common measurement approaches with healthcare teams**^**a**^		
			**Data source**	**Operationalization**	**Specificity**
**Direct**	***Pre-task***	*Shared mental model measures* [21, 22] In one pre-task mental model measure, surgical laparotomy team members [21] sorted 20 tasks (e.g. insert urinary catheter; close incision) based on when (and by whom) they should be performed, using an online programme	After coming to learn of the simulation, participants privately report their expectations of the team, task, or situation	*Teammate similarity*: Combining all individual responses to calculate an index that reflects variability in responses across members of a team *Team or individual level accuracy*: Extent to which team average or individual responses deviate from standards or expectations (i.e. rated by an expert)	Sometimes event-specific: Incorporates tasks, member roles, or key information in specialized situations. Such tools often developed through consultation with experts with a specific procedure Commonly context-specific: Designed to examine perceptions within specific medical specialties and tasks
	***In-task***	*Situational Awareness Global Assessment measures* [23, 24] Several measures are adapted for use with trauma team members (e.g. leader, nurse, airway manager) who privately complete surveys during freezes. Brief measures incorporate multiple-choice and open-ended items, such as the following: • ‘What equipment may you need in the next few minutes?’ [23] • ‘Who is the team leader at this point?’ [24]	Within the task, participants pause to privately complete items reflecting their understanding of the situation, task, or team		
	***Post-task***	*Post-task team situational awareness measures *[4] Members complete multiple-choice items after the task to rate the most likely next steps in treatment: ‘After getting pulses back, the team should prioritize the administration of which medication? [participant selects from list]’ [4]	After the task, participants privately complete items regarding their understanding of the preceding situation/task or the next steps for the team		
**Indirect**	***Pre-task***	No specific pre-task measures identified		*Team process*: Items aggregated to reflect members’ and observers’ perceptions (e.g. how much team perceived to have shared understanding) *Individual ratings*: Observers evaluate skills or actions of individual *Behaviour frequencies*: Observer tallies frequency of team behaviours during entire simulation *Behaviour patterns*: Observer ratings analysed to identify combinations of behaviours related in time	Rarely event specific: Not uniquely designed for scenarios or tasks Often context specific: Designed for specific medical specialties and types of team tasks Can be general to context and event: Capturing perceptions of teams across settings (i.e. needs no adaptation regardless of setting)
	***In-task***	*Situational awareness rating systems* [25–27] Observers code for behaviours that signal situational awareness, including behaviours like the extent that leaders seek input (e.g. request information and invite others to provide information) [27] or members’ use of closed-loop communication (e.g. call-out, check-back) [26]	Momentary coding of leader/follower behaviours or team states, using video or in vivo observer code guides		
	***Post-task***	*Measures of nontechnical skills* [18, 28] Observers evaluate individual/team tendencies (e.g. ‘members gathered information and were anticipatory’) [28] *Teamwork and team process measures* [29–33] Observers or team members reflect on team processes (‘How would you characterize your team’s shared understanding of the clinical scenario?’) [4] or specific components (e.g. ‘Each team member demonstrated clear understanding of their role’) [29]	Post-task surveys, completed by members and/or observers, often reflecting on multiple aspects of team/individual performance		

^a^See Online Supplemental Material for detailed description of all example measures listed in Table 1

While team cognition measurement in healthcare simulation and education literature is overrepresented by indirect post-task tools, we did identify direct measures that are used before or during simulation. In the following sections, we will explore the merits and operationalization of direct and indirect assessment as well as the role of measurement timing as it pertains to the evolution of team cognition during performance.

### Applying directness and temporality to existing literature

Our choice to highlight direct and indirect dimensions reflect the objective and subjective distinction offered by Endlsey [22], who characterized objective (direct) measures as gold standard tools relative to subjective (indirect).^1^ Direct measures integrate probes that are delivered pre-task (e.g. each member privately indicates their understanding of the task and the roles they and each member of the team play), in-task (e.g. each member privately indicates specifics relevant to the patient condition and the tasks ahead), and post-task (e.g. each member privately indicates the management plan at the time of task completion). Designing these probes is often demanding, requiring adaptation for specific tasks using input from experts to identify key aspects of the team or task that require members to gain accurate and shared understanding [22]. Responses are then aggregated and assessed relative to their similarity with one another (sharedness) or to a prospectively identified standard (accuracy). This means that analyses are critical to operationalize team cognition that is assessed directly, because calculations are needed to convert individual responses into estimates of agreement across members.

The archetypal tool for direct measurement of situational awareness is the Situational Awareness Global Assessment Tool (SAGAT) [15]. Three studies we identified used such an approach to examine how accurate and similar members were in their awareness of next steps in patient management [4, 28, 29]. These direct measures have previously been dismissed by some authors as intrusive in healthcare simulation [4, 31, 37], but despite these concerns, there is no empirical evidence that these intrusions influence training fidelity [22], and two studies of healthcare teams which utilized these tools reported no adverse effect from their utilization [28, 29]. Direct assessment for team mental models typically occurs via a survey delivered outside of a specific task or in the pre-task phase. We found two studies in the healthcare literature [26, 27] that include such tools to replicate, as nearly as possible, the actual content of an individual’s cognitions about the relevant task or their beliefs about teamwork. For instance, Burtscher and colleagues [27] surveyed experts to construct a list of 30 tasks (e.g. ventilate patient, reposition head) that every member sorted based on their priority during a procedure and the team members who are responsible for each task. Analyses were subsequently used to estimate sharedness and accuracy of responses.

Direct measures can be resource intensive to develop, lack feasibility in some settings, and have limited generalizability between contexts [12, 20], but these tools tap into the content and structure of individual and team cognition and are the only unmediated way to capture concepts like sharedness of cognition. Whereas in-task direct measures are central for this aim, the online materials associated with this article also highlight underused direct measures that assess members before or after the task, which can be utilized when intrusiveness remains a concern (see also the next section on temporality).

Indirect measures for team cognition represent the dominant form of measurement in healthcare teams research. These measures use proxies including behaviours or communication patterns that are theorized to reflect or promote sharedness. Many indirect measurement tools identified in recent systematic reviews represent composite non-technical skills assessments derived from four initial tools that have been adapted for varying medical domains. This finding is most striking in a recent review by McMullan and colleagues [7], who identified that out of the 88 studies that used communication measures within surgical settings, 22 included the original Non-TECHnical Skills (NOTECHS) [38] or variant tools, 20 were derived from the original Anesthetists’ Non-Technical Skills (ANTS) [25] measure, and 20 were based on Non-Technical Skills for Surgeons (NOTSS) [18]. As aggregate team process assessments, these tools have varying emphasis and often infer team cognition from observed processes, such as by evaluating the frequency of communication or efforts of leaders to share information.

While the above tools are favoured in simulation due to their ease of use, their indirect assessment of representations means they offer limited ability to assess the sharedness and accuracy of team cognition. There may, for instance, be several ‘causes’ for a group to not engage in a specific set of behaviours. For instance, a group with extremely high sharedness might appear to be a ‘silent group’ and may not engage in much sharing behaviour. Such ‘silent’ group members may instead have highly efficient communication, whereby information is infrequent but meaningful.

There are certainly circumstances under which indirect measurement is ideal or at least complimentary to direct measurement. Below we will elaborate further on examples of indirect tools specifically designed for team cognition processes, which can result in more actionable feedback for simulation participants.

#### When are assessments gathered?

In addition to the underlying direct or indirect dimension, *when* data is collected has a significant impact on the insights a tool can offer about team cognition. Many team cognition tools identified in recent reviews are post-task measures where an observer or team members document observations or reflections immediately after a team performance. The framework presented in Fig. 1 highlights opportunities for direct and indirect measurement to be completed by team members or observers before, during, and after the task.

Many of the observational tools in the healthcare teams literature are performed post-task and include an aggregate assessment of non-technical skills, which include team cognition. These tools are limited by their reliance on retrospective assessment and limited evaluation of processes specific to team cognition development and maintenance. In contrast, there are emerging observational tools which collect and code behavioural data continuously throughout team performance which may be more effective at identifying team cognition processes. As one example, the Team Reflection Behavioural Observation (TuRBO) System [31] uses continuous momentary coding for observers of recorded team performances to identify when and how participants engage in reflection behaviours. Despite its indirect approach, this type of assessment has the capacity to offer rich insights due to an emphasis on team cognition processes and by monitoring the evolution of these processes throughout performance.

#### What concepts are targeted?

In an effort to maintain generalizability, our framework does not distinguish tools across the specific content or concept of interest for a measure, but this is an important dimension to consider. Many common measures contain broad, unidimensional assessments or claim to capture specific constructs such as situational awareness while actually evaluating team behaviours like communication and leadership or processes like coping and decision-making [1]. As one example spanning both of these concerns, the original Anesthetists’ Non-Technical Skills tool operationalizes situational awareness through a single-item observer rating regarding the extent leaders gather information, recognize key situations, and are anticipatory [25].

Emerging advances in healthcare research have attempted to provide more precision. For instance, O’Neill and colleagues' [30] measure involves a coding tool where observers identify moments in time when members demonstrate behaviours reflecting one of seven dimensions of situational awareness. Such measures, while indirect, are studied specifically with team cognition in mind and collect data continuously during a performance. This type of measure offers a more thorough understanding of key team behaviours that underpin the development and maintenance of team cognition and can provide actionable feedback for performers.

## Discussion

Below, we provide simulation educators with a road map to apply team cognition assessments that better align measurement with their training objectives. We then highlight opportunities for researchers to revolutionize healthcare team cognition measurement by exploring the applicability of these tools in healthcare simulation and integrating advances made in human factors and organizational psychology.

### For the educator: optimize measurement to inform skill development

Fernandez and colleagues [1] highlighted seven recommendations for integrating team cognition within simulation programmes; one recommendation was to focus on measurement as an essential tool to document the effectiveness of training while enhancing the feedback used in debriefing [1]. When educators identify a specific feature of team cognition to assess, and deliberately assess with appropriate timing and sources for data, they are more likely to offer trainees opportunities for richer discussion and more actionable feedback. Below we outline an evaluation approach that highlights pre-task, in-task, and post-task assessments to support a debriefing and training model with the precision to inform training and optimize desired actions.

#### Pre-task direct assessment

Pre-task assessment is an important and underexplored gap in the simulation literature. Team situational awareness cannot be captured prior to an ad hoc team performance, as there are neither any team interactions nor a team ‘situation’ of which to be aware. Pre-task measures are, however, a powerful tool to evaluate members’ static perceptions of the group task and relative contributions to it at the outset of the task or, rather, their mental models. We recommend integrating pre-task measures such as the card sorting or concept mapping activities in the online supplemental material, which could include a paper- or tablet-based survey completed after the initial details of the case have been provided but before the team briefing. The elicited data can then be assessed for sharedness and accuracy at the team level and used to inform debriefing regarding how the team leveraged actions to identify and overcome discrepancies as their shared understanding evolved throughout the management of the case. Pre-task tools allow a baseline assessment of team member mental models of the case to map their evolution over time while also informing actions that are observed throughout the case.

#### In-task direct and indirect assessment

In-task probes offer direct insight into member cognitions (representations), while behavioural coding offers a continuous assessment of member communication and action (processes). Mechanisms for direct in-task data collection can occur through periodic pauses for brief questionnaires, such as SAGAT [15] or similar variants [40]. To date, such measures have not been readily adopted in healthcare teams simulation, yet their capacity to inform an understanding of team cognition and thereby support debriefing represents an important opportunity for healthcare educators. There are also recently-described indirect in-task behavioural coding tools. These measures involve having observers track member behaviours or rate interactions across moments [30, 31] and may compliment direct assessment in a way that can calibrate the processes observed by the facilitator to the emergent representations held in the minds of the team.

#### Post-task direct and indirect assessment

Evaluating the state of team cognition upon completion of the team’s performance represents an important opportunity to correlate what team members are thinking to how they performed. Responses from observers and team members can integrate indirect assessments of recalled behaviour and performance with perceptions of sharedness amongst members. We also recommend post-task direct measures of situational awareness to evaluate the extent that members gained similar understanding of the ongoing situation and of next steps in patient management. As one example that integrates both direct and indirect post-task measures is Rosenman and colleagues’ [4] post-task probe assessing similarity in member projection states about subsequent treatment (i.e. direct assessment of sharedness), along with a post-task survey assessing team member perceptions of the team’s situational awareness (i.e. indirect assessment). In this case, perceptions of team cognition (the indirect item) included the survey question ‘How would you characterize your team’s shared understanding of the clinical scenario?’ [4]. While the authors reported that the direct and indirect measures of situational awareness were positively correlated, only the direct measure predicted team clinical performance.

### For the researcher: develop tools with potential for wide reach and high adoption

An increasingly translational research programme within healthcare team simulation requires developing direct measurement tools with higher ease of use. It is a resource-intensive process for simulation educators to conduct task analyses that identify key team tasks and cognitions to assess for a specific context or situation [21], and this may explain why many of our present tools are brief observer reports. There are two potential pathways to address this issue. First, researchers could innovate and develop tools that are transferable across contexts — measures could target features of team cognition that are present in multiple contexts, reducing the need to adapt tools with task analysis. Second, researchers could better articulate how their tools could be adapted by educators. Like the training materials that accompany other training-focused team measures such as NOTECHs [17], researchers who develop team cognition tools that require task analysis could provide training materials regarding how to develop new items for novel tasks or simulation scenarios. Regardless of which of the above steps is most feasible, translation into simulation-based healthcare teams training also demands evidence that more precise tools relate to training outcomes. Given that team cognition assessment is itself an educational intervention, researchers should examine the extent to which direct measurement of team cognition impacts team member behaviours by inviting them to reflect in the moment. Other areas of enquiry include an assessment of how direct measurement generates a richer debriefing session, further reflection, or enhanced team functioning in subsequent sessions.

Organizational psychology and human factors scholarship also offers insights into additional opportunities to advance research involving indirect measures. Recently completed reviews by Mathieu et al. [19] and Mohammed et al. [14] have highlighted that the future of team cognition research needs to address assessment methods that emphasize the temporal and dynamic nature of team cognition. The methods these authors propose are intended to be non-invasive tools aimed at generating metadata, which can then be analysed automatically by techniques such as computer-aided text analysis. One emerging technique highlighted by Mathieu et al. [19] is the use of wearable technology that tracks the content and tone of team member communication while also tracking their movement within the space and position relative to one another. Such techniques are, indeed, indirect tools of team cognition because they focus on what members ‘do’ rather than their sharedness or accuracy of cognitions. Yet, they may prove particularly valuable in those circumstances when in-task probes or pre-task questionnaires are inappropriate (e.g. live resuscitation) or to map the evolution of team member behaviours between in task probes.

## Conclusion

Team mental models and team situational awareness are core terms within the healthcare simulation lexicon, but they suffer from inaccurate definition and indirect assessment when measured as a component of teamwork. Advances in the fields of human factors and organizational psychology have established the importance of direct measurement to inform evaluation of team cognition and tools for such measurement have been successfully translated into the healthcare team simulation sphere; however, they represent the minority amongst a plethora of indirect and imprecise measures. We offer a novel framework for team cognition assessment in simulation and identify several key dimensions that distinguish ‘types’ of measures. Efforts to train for team cognition would benefit from more precise language surrounding these constructs and the integration of more direct measures of team cognition that capture assessments across key temporal phases. To support a more robust understanding about collaborating in ad hoc healthcare action teams, we hope that simulation facilitators and researchers will use our findings to refine their vocabulary and adopt more deliberate measures that align with emerging theories of team cognition from human factors and organizational psychology.

## Supplementary Information

Additional file 1. Supplemental Online Material. Simplified Description of Selected Direct and Indirect Team Cognition Measures [4, 18, 32,33,34,35,36,37,25,26,27,28,29,30,31].

## Data Availability

No datasets were generated or analysed during the current study.

## Abbreviations

SAGAT: Situational Awareness Global Assessment Tool
NOTECHS: Non-TECHnical Skills tool
ANTS: Anesthetists Non-Technical Skills tool
NOTSS: Non-Technical Skills for Surgeons tool
TuRBO: Team Reflection Behavioural Observation tool

## Footnotes

1 We use the terms indirect and direct because this dichotomy more accurately addresses the mechanism of measurement — ‘directly’ accessing cognition through members’ responses, as opposed an indirect view mediated through member behaviour or the team’s performance. There is likely no truly ‘objective’ measures of team cognition constructs, so labels of objective and subjective are less accurate in a literal sense.
