# Supplementary material for: Team cognition in healthcare simulation: a framework for deliberate measurement
Source: Adv Simul (Lond). 2025 Mar 24;10:12. doi: 10.1186/s41077-025-00333-7 (PMC11931873; doi:10.1186/s41077-025-00333-7)
Supplement: Supplementary file 1 — Additional file 1. Supplemental Online Material. Simplified Description of Selected Direct and Indirect Team Cognition Measures [4, 18, 32333435363725262728293031]. [file 41077_2025_333_MOESM1_ESM.docx]

**Supplemental Online Material.**

| **Simplified Description of Selected Direct and Indirect Team Cognition Measures .**^4,18,25–37^ | | | | |
| --- | --- | --- | --- | --- |
| **Tool / Citation**  **Operationalization** | **Timing and data source** | **Level of analysis** | **Context specificity** | **Example item** |
| **DIRECT TOOLS perhaps colour block these and other boxes for transparency of section breaks?** | | | | |
| **Shared Mental Models**^26^  Members of surgical teams sorted 20 tasks based on when during the procedure they should be performed (ordering). | **Pre-task**  Each member completed the activity privately, prior to beginning simulation. | **Team similarity**  Team similarity computed based on similarity in responses. | **Context/event-specific**  Measure was developed by consulting experts to identify 20 tasks required for surgical laparotomies. | Participants receive list of 20 tasks (e.g., insert urinary catheter; close incision) and orient them within online program based on team member roles and temporal order. |
| **Shared Mental Models**^27^  Anaesthesia residents and nurses completed a concept mapping task prior to simulation on a whiteboard, attributing based on member roles and temporal order. | **Pre-task**  Each member completed the activity privately, prior to beginning simulation. | **Individual & team similarity/accuracy**  Individual accuracy via comparisons with expert feedback. Team similarity computed based on similarity in responses. | **Context/event-specific**  Measure was developed by interviewing ten experts to identify ten ‘concepts’ relevant to induction of general anesthesia. | Participants receive list of 30 tasks (e.g., ventilate patient, reposition head, announce drug administration) and place them onto whiteboard based on team member roles and temporal order. |
| **Team Situation Awareness Global Assessment Technique**^28^  **Situational awareness**  Interprofessional trauma teams (Trauma team leader, Nurse, Airway manager) completed written assessments during three planned freezes within simulation. | **In-task**  Members provide written responses to eight open-ended items during three ‘freezes’ in simulation. | **Sum of individual accuracy**  Added individual members’ situational awareness accuracy, based on similarity to expert ratings. | **Context/event-specific**  Task analysis conducted to identify major goals of simulation task, which then were included in measure. | Example items:  “What is contributing to the patient’s airway compromise?”  “What equipment may you need in the next few minutes?” |
| **Situation Awareness Global Assessment Technique – Pediatric Care**^29^  **Situational awareness**  Interprofessional pediatric trauma teams (Nurses, Resident, Consultant) completed written assessments during one freeze 10 minutes into the session, and another at the completion of simulation. | **In-task**  Members provide written responses to items during ‘freezes’ in simulation: (a) 8 multiple-choice items, (b) one open-ended diagnosis item, and (c) one Likert-type rating for the quality of teamwork. | **Team similarity**  Computed team-level similarity at a descriptive level (% agreement among members) | **Context/event-specific**  Task analysis conducted to identify major goals of simulation task, which then were included in measure | Example items:  “What is the patient’s HB count?” [a. < 5 mmol/L; b. 5–7 mmol/L; c. > 7 mmol/L; d. I do not know]”  “Who is the team leader at this point?”  Add quotations at end of Qs? |
| **Team Situational Awareness survey**^4^  **Situational awareness**  Members complete multiple choice items after the task to rate the most likely next steps in treatment. | **Post-task**  Each member completed items about future group actions. | **Team similarity, individual accuracy**  Compute similarity across members, and each member’s deviation from group responses. | **Context specific, event specific**  Measure was developed by experts to reflect decisions or contingencies within the simulation scenario. | Example item:  “After getting pulses back the team should prioritize the administration of which medication? [participant selects from list of medications].” |
| **INDIRECT TOOLS perhaps colour block these boxes for transparency?** | | | | |
| **Situational awareness.**^30^  Observers rate teams on the presence or absence of behaviours that are aligned with seven dimensions of situational awareness (e.g., anticipate and plan, prioritize attention, reassess patient). | **In-task**  Simulation video recordings broken into key 3-minute segments, and behaviour was rated after watching each segment. | **Team behaviour frequency**  Observers score observed behaviors for each dimension of situational awareness. | **Context-specific, event general.**  Tool used during simulation feedback and debriefing intervention, but could be used in similar settings. | Example behavior codes supporting anticipation planning:  Using “If/Then” statements (“If the patient goes apnoeic, then we will need to intubate”) |
| **TuRBO System**^31^  **Reflection**  Observational rating scale tracking conscious reflection during ongoing task based on the duration of time spent updating shared understanding. | **In-task**  Observers review video and code the time during which reflection behaviours are evident. | **Team behaviour frequency**  Computing the minutes members engaged in reflection types. | **General tool**  Applicable across settings in which video can be acquired, and where team reflection is viable. | Example code: Members coded as ‘actively seeking input’ when they request information and invite others to provide information. |
| **Non-technical Skills for Surgeons**^32^  **Situational awareness**  Rating surgeon behaviours that support situational awareness. | **In-task.**  Observer used video to rate each communication incidence between surgeon and others. | **Individual behaviour frequency**  Observer rates the behavior; behavioural instance is analyzed alongside other momentary data. | **Context-specific, event general.**  Designed for evaluation of surgeons. | Manually code behaviour, such as instances of closed-loop communication (e.g., call-out, check-back) |
| **Anesthetists’ Nontechnical Skills**^25^  **Non-technical skills**  **Situational awareness**  Rating scale of individuals across four categories (i.e., cooperation, leadership, situation awareness, decision making). | **Post task**  Observer rates participants following task. | **Individual behaviour quality**  Anesthesiologists rated individually. | **Context- specific, event general**  Tool is designed for anesthesiology. | For situational awareness, individuals rated on the extent they gather information, recognize key situations, and are anticipatory |
| **Non-Technical Skills for Surgeons**^18^  **Nontechnical skills**  Surgeon or surgical trainee behaviours that develop shared understanding (i.e., gathering information, anticipating future state, establishing shared understanding). | **Post-task**  Observer creates a summative rating for each category at the end of the task. | **Individual behaviour quality**  Observer rates the surgeon or surgeon-trainee subjectively, based on key behaviours | **Context-specific, event general**  Designed for evaluating surgical trainees across situations. | Examples of gathering information item: “Ensures that all relevant investigations have been reviewed and are available.” |
| **Mayo High Performance Teamwork Scale**^33^  **Shared understanding**  Team members rate group using 16 teamwork behaviours – with several items relating to shared understanding. | **Post-task**  Members use a scale to rate behaviour frequency from never/rarely to consistently. | **Team behaviour quality.**  Each participant completed the scale reflecting the whole team. | **General tool**  Applicable to many settings. | Example items:  “Each team member demonstrates clear understanding of their role.” |
| **Clinical Teamwork Scale**^34^  **Shared understanding**  A rating scale using descriptive anchors for several teamwork behaviours, which include items reflecting situational awareness and shared mental models (e.g., decision-making out loud to establish sharedness; establishing leader and follower roles). | **Post-task**  Trained observers review video and at the conclusion of the review, rate each of the behaviour types. | **Team behaviour quality**  Observers rate behaviors of teams as a whole using behavioral anchors. | **General tool**  Applicable to many settings. | Example item reflecting transparent thinking:  “The team members use “think out loud” communication so that all team members share the same mental model of the situation” |
| **Team Emergency Assessment Measure**^35^  **Situational awareness**  Resuscitation teamwork assessment including nine dimensions, with situational awareness and decision making as one category. | **Post-task**  Observers rate subjective evaluations of team after completion of task using 11 items. | **Team behaviour quality**  Observer rates group interactions. | **General tool**  Designed for the emergency medicine, but items are general in nature. | Example item: “The team anticipated potential actions.” |
| **Ottawa CRS**^36^  **Team processes**  Observer rates individual behaviour (e.g., problem solving, situational awareness, resource utilization). Three items capture situational awareness. | **Post task**  Observer subjectively rated each resident trainee. | **Individual behaviour quality**  Each team member is rated. | **Context-specific, event general**  Designed for resuscitation task | Example situational awareness item: “Becomes fixated easily despite repeated cues” |
| **OSCAR**^37^  **Team processes.**  Subjective evaluations of individuals on a scale spanning several dimensions, several of which reflect situational awareness. Scores distinguished into subgroups (i.e., Anaesthetist, physician, nurse). | **Post-task**  Observer rates evident behaviours. | **Sub-group behaviour frequency**  Observers complete ratings of behaviours initiated by members of three interprofessional team subgroups. | **Context-specific, event general.**  Used for varying procedures requiring interprofessional teams. | Example behavioural codes for ‘monitoring’: Physician checks ventilation adequately/Anaesthetist monitors progress of resuscitation protocol/ Nurse monitors patient dignity. |
| **Situational awareness**^4^  Item completed to report sharedness in mental models. | **Post-task**  Each member completes single Likert-type item after task. | **Individual & team similarity perceptions**  Measures individuals perceived sharedness, or the team mean. | **General tool**  Applicable to many settings. | “How would you characterize your team’s shared understanding of the clinical scenario?” |
| *Note. Table is clustered based upon indirect and direct dimensions as well as: (a) operationalization, or what the developers intended the measure to capture, (b) the timing of data collection, (c) the extent that measures produce estimates for each member and/or the entire team, (d) the extent that measure was designed specific to a domain of medicine (e.g., surgery, anesthesiology) and/or for a specific task.* | | | | |
